# Clinical Characteristics, Risk Factors, and Outcomes of Acute Pulmonary Embolism in Asian Population

**DOI:** 10.3390/jcm11236954

**Published:** 2022-11-25

**Authors:** Chaiwat Bumroongkit, Athavudh Deesomchok, Chalerm Liwsrisakun, Chaicharn Pothirat, Theerakorn Theerakittikul, Atikun Limsukon, Konlawij Trongtrakul, Pattraporn Tajarernmuang, Nutchanok Niyatiwatchanchai, Arintaya Phrommintikul, Tul Chaikitmongkol, Juntima Euathrongchit, Chartaroon Rimsukcharoenchai, Juthamas Inchai, Warawut Chaiwong

**Affiliations:** 1 Division of Pulmonary, Critical Care, and Allergy, Department of Internal Medicine, Faculty of Medicine, Chiang Mai University, Chiang Mai 50200, Thailand; 2 Division of Cardiology, Department of Internal Medicine, Faculty of Medicine, Chiang Mai University, Chiang Mai 50200, Thailand; 3 Department of Radiology, Faculty of Medicine, Chiang Mai University, Chiang Mai 50200, Thailand; 4 Division of Cardiovascular and Thoracic Surgery, Department of Surgery, Faculty of Medicine, Chiang Mai University, Chiang Mai 50200, Thailand

**Keywords:** acute pulmonary embolism, clinical characteristics, risk factor, mortality, the Asian population

## Abstract

Background: Acute pulmonary embolism (APE) is a common condition with increasing worldwide incidence. However, the clinical characteristics, risk factors, and clinical outcomes of APE in the Asian population especially in the Thai population are still limited. Therefore, the objective of this study was to identify the clinical characteristics, risk factors, and clinical outcomes of APE in the Asian population. Methods: A cross-sectional study was conducted on patients diagnosed with APE at Chiang Mai University Hospital, Thailand during 2011–2020. Results: During the study period, 696 patients confirmed the diagnosis of APE with a mean age of 57.7 ± 15.7 years and 41.1% males. APE was suspected in 468 of 696 patients (67.2%), while 228 patients (32.8%) had incidental PE. Active malignancy during treatment was found in 388 (55.7%). Dyspnea, cough, and chest pain were the most common presenting symptoms. Respiratory failure was found in 129 patients (18.6%). The thirty-day all-cause mortality rate was 19.1%. PE-related mortality was 5.6%. Most PE-related mortality was high-risk PE. Conclusion: APE was not uncommon in the Asian population. Active cancer, especially lung cancer was the most common risk factors. High-risk and intermediate-high-risk PE were associated with high mortality. Risk stratification and prompt management are warranted to improve outcomes.

## 1. Introduction

Acute pulmonary embolism (APE) is a common condition and increasing incidence in Western countries [1,2] and has high morbidity and mortality without appropriate management [3,4]. In Asian countries, the incidence of APE was lower than in Western countries [5,6], might be from low awareness of clinical suspicion and limited healthcare [7]. APE is known as *‘the great masquerader’* because the clinical manifestations including symptoms, signs, and investigations were nonspecific and varying from asymptomatic (incidental PE) to acute respiratory failure and/or shock (high-risk PE) [8]. There are many clinical risk factors associated with venous thromboembolism including patient factors such as increased age, co-morbidities, thrombophilia, obesity, cancers, inflammatory bowel diseases [9,10], and history of venous thromboembolism; and situation factors such as surgery, trauma, and immobilization. The increasing availability of newer imaging modalities is likely to improve the diagnosis. Despite the advance in diagnostic methods, delays in APE diagnosis are still common [11] and affect the worse clinical outcomes [12]. A high index of clinical suspicion, appropriate diagnostic investigations and clinical severity assessment, and appropriate therapy are crucial for the management of APE. However, the clinical characteristics, risk factors, and clinical outcomes of APE in the Asian population especially Thai are still required. Therefore, the objective of this study is to identify the clinical characteristics, risk factors, and clinical outcomes of patients with APE in our center which could represent APE in the Asian population.

## 2. Materials and Methods

### 2.1. Study Design

This cross-sectional study was approved by the Research Ethics Committee of the Faculty of Medicine, Chiang Mai University (Institutional Review Board (IRB) approval number: MED-2564-08294, date of approval; 29 July 2021) in compliance with the relevant international ethical guidelines, laws and regulations including Declaration of Helsinki. The Research Ethics Committee allowed a waiver of informed consent because the data were reviewed retrospectively from the medical records.

### 2.2. Subject Selection

All patients with the age 15 or more at Chiang Mai University Hospital, Chiang Mai, Thailand during the year 2011 to 2020 with ICD-10 coding I26.0 (pulmonary embolism with mention of acute cor pulmonale) and I26.9 (pulmonary embolism without mention of acute cor pulmonale) were recruited. Medical records were reviewed to confirm the diagnosis of APE and only patients who had confirmed the diagnosis of APE proceeded for data analysis.

The diagnosis of APE was confirmed by radiologists, in which thrombus was demonstrated in the pulmonary arteries and its branches by computed tomographic pulmonary angiography (CTPA) or CT chest with contrast.

Patients with APE were classified along with the 2019 European Society of Cardiology (ESC) guideline [8] as high-risk PE if there was evidence of hemodynamic instability (defined as systolic blood pressure [SBP] < 90 mmHg for >15 min, drop in SBP of 40 mmHg or more from baseline or requiring hemodynamic support) or cardiac arrest, and intermediate-risk or low-risk PE if there was no hemodynamic instability. The predisposing risk factors were identified.

Patients’ data, including demographics, symptoms, signs within the first 24 h of diagnosis, risk factors, co-morbidities, investigations (including electrocardiogram, chest radiography, cardiac biomarker, blood lactate, D-dimer, oxygen saturation, echocardiogram, CTPA, Wells score, Pulmonary Embolism Severity Index (PESI) and 2019 ESC risk stratification were recorded. Treatments and outcomes at one month were also reviewed.

### 2.3. Statistical Analysis

Continuous data were expressed as mean with standard deviation (SD) or median with interquartile range (IQR) as appropriate. Categorical data were expressed as frequencies and percentages. Independent samples *t*-tests or Mann-Whitney U Test was used to analyze the differences between the survivor and non-survivor groups for parametric and non-parametric data, respectively. Fisher’s exact test was used to compare the categorical data between groups. Statistical significance was accepted at a *p*-value < 0.05. All statistical analyses were performed using STATA version 15 (StataCorp, College Station, TX, USA).

## 3. Results

### 3.1. Patient Characteristics

**Figure 1:**
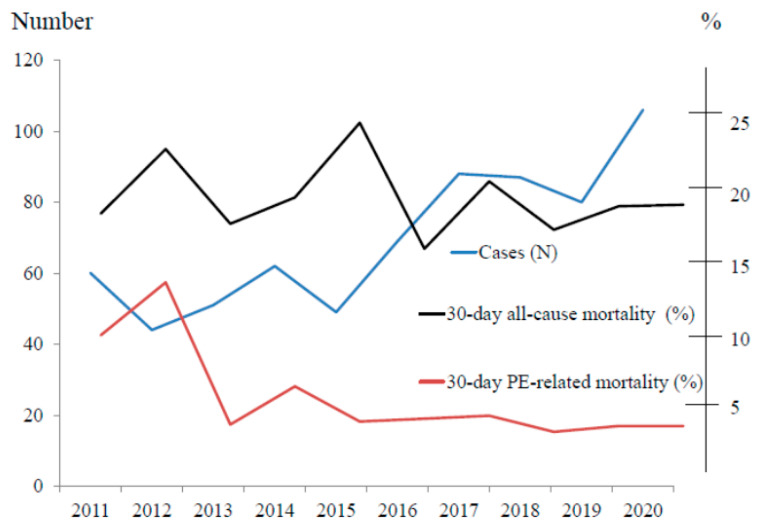
Number of APE Cases (*n*) and Mortality Rate (%) during 2011–2020.

During 2011–2020, a total of 1560 patient records were recruited from the ICD-10 coding diagnosis and the number of APE patients tended to increase over time (Figure 1).

Eight-hundred and sixty-four patient records were excluded due to age under 15 years, non-Asian ethnicities, repeated cases, and no CT confirmation of diagnosis. Six-hundred and ninety-six patients were analyzed with a mean age of 57.7 ± 15.7 years (range 15–98) and 41.1% male. Three-hundred and eighty-eight patients (55.7%) had at least one co-morbidity and hypertension was the most common. Deep vein thrombosis (DVT) was diagnosed together with APE in 245 patients (35.3%). Four-hundred and sixty-eight patients (67.2%) had clinically suspected APE, while 228 patients (32.8%) were incidental PE. Forty-five patients (6.5%) have previous VTE in the past. Other patient characteristics and co-morbidities were demonstrated in Table 1.

### 3.2. Risk Factors

Risk factors for APE were found in 560 patients (80.5%). Active malignancy was the most common risk factor (388 patients, 55.7%) followed by immobilization (211 patients, 30.4%) and surgery or trauma required endotracheal intubation or epidural anesthesia within four weeks (141 patients, 20.3%). Lung cancer, cholangiocarcinoma, and colorectal cancer were common in cancer-associated APE. Adenocarcinoma was the most common cell type (147 patients, 70.3%) and distant metastases were found in 282 patients (72.7%). Other risk factors and the detail of cancer associated with APE were presented in Supplementary Tables S1 and S2.

### 3.3. Clinical Presentation and Severity

Dyspnea either at rest (65.3%) or on exertion (39.5%), cough (23.3%), and chest pain (16.5%) were the most common presenting symptoms, while low peripheral capillary oxygen saturation (SpO_2_) at room air (48.3%), tachycardia (46.9%) and tachypnea (22.7%) were the most common physical findings. Hemoptysis was found in only 5.5%. 18.6% of patients with APE presented with respiratory failure and required invasive mechanical ventilator support, while 11.9% of patients had low blood pressure (<90/60 mmHg). Extremities swelling suggestive of DVT was found at 18.3% and pain on limb palpation was found at 5.8%. Other symptoms and physical findings were demonstrated in Table 2.

Electrocardiogram (ECG) and chest X-ray (CXR) findings were shown in Supplementary Tables S3 and S4. The most common abnormal ECG findings were sinus tachycardia (56.5%), followed by the pattern of S1Q3T3 (23.0%) and T wave inversion at V1–V4 which reflect RV strain pattern (16.1%), while PE related CXR findings; oligemic or Westermark sign was found 14.4%, cardiomegaly 11.8%, prominent central pulmonary trunk 7.6% and Hampton hump 6.6%. Normal CXR finding was found in 47.1% of patients with APE.

CT findings of APE were shown in Supplementary Table S5. Clots in the mediastinal (central) level of pulmonary arteries including the pulmonary trunk, main pulmonary, and descending pulmonary artery were found in 310 patients (44.5%). The pulmonary infarction was found in 21.8%. CT signs of right ventricular (RV) pressure load were also presented in Supplementary Table S5. Ultrasound DVT was done in 292 patients and 160 patients (54.8%) had concomitant DVT which the popliteal vein (56.9%) was the most common site. Echocardiography was done in 332 patients and RV dysfunction was demonstrated in 132 patients (39.8%). Other pertinent investigations related to PE including serum biomarkers (troponin-T, D-dimer, blood lactate), ultrasound DVT, and echocardiography were shown in Supplementary Table S6.

The severity of APE by Pulmonary Embolism Severity Index (PESI) scores was classified into Class I (62 patients, 8.9%), Class II (98 patients, 14.1%), Class III (191 patients, 27.4%), Class IV (151 patients, 21.7%) and Class V (194 patients, 27.9%). The patients were also classified risk according to 2019 ESC risk stratification as high-risk PE (78 patients, 11.2%), intermediate-high-risk PE (96 patients, 13.8%), intermediate-low-risk PE (376 patients, 54.0%) and low-risk PE (146 patients, 21.0%).

### 3.4. Treatments and Outcomes of APE

Treatments and outcomes of APE are shown in Table 3 and Table 4. For the acute treatment, 403 patients (57.9%) received low molecular weight heparin (LMWH), 127 patients (18.2%) received unfractionated heparin (UFH), 104 patients (14.9%) received UFH and then switched to LMWH. Thrombolytic therapy was given in 37 patients (5.3%) and surgical thromboembolectomy was done in 12 patients (1.7%). For long-term treatment, 252 patients (36.2%) received warfarin, 247 patients (35.5%) received LMWH, and 40 patients (5.7%) received direct oral anticoagulants (DOACs). In our study, there were 25 patients (3.6%) who did not get any specific treatment due to the presence of contraindications to anticoagulant and thrombolytic therapy and nine patients (1.3%) denied treatment. Rescue medical thrombolytic therapy was needed in four cases and rescue surgical thromboembolectomy was performed in four cases.

**Figure 2:**
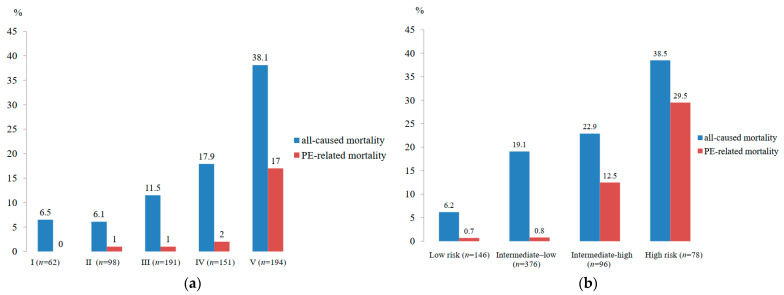
Thirty-day All-cause Mortality and PE-related Mortality According to PESI Classification and ESC 2019 Risk Stratification. Note: (**a**), 30-day all-cause mortality and PE-related mortality according to PESI classification; (**b**), 30-day all-cause mortality and PE-related mortality according to ESC 2019 risk stratification.

The thirty-day all-cause mortality rate was 19.1% (133 patients), and PE-related mortality was 5.6% (39 patients). 59% and 30.8% of PE-related death were high-risk PE (*n* = 23) and intermediate-high-risk PE (*n* = 12), respectively. The thirty-day all-cause mortality rate was 40.0% (10/25 patients) and 18.3% (123/671) in subjects with anticoagulated and those with have not received anticoagulation due to contraindications, respectively. The thirty-day PE-related mortality rate was 8.0% (2/25 patients) and 5.5% (37/671) in subjects with anticoagulated and those with did not get anticoagulation, respectively. Sepsis was the most common cause of non-PE-related death (37 of 94 patients, 39.4%). The higher PESI class and 2019 ESC risk class are associated with higher mortality as demonstrated in Figure 2 (PE-related mortality rate was highest in the PESI class V and 2019 ESC high-risk). Major bleeding was found in 57 patients (8.2%). The clots were not resolved in 25 patients (3.6%) and turned later to chronic thromboembolic pulmonary hypertension (CTEPH). More data are shown in Table 4. We also observed that the PE-related mortality rate tended to decrease over time during the study period (Figure 1).

## 4. Discussion

Acute pulmonary embolism is not rare as thought in the Asian population. From the population-based study by Lee, et al. [6], the annual symptomatic venous thromboembolism (VTE) incidence in Korea, Taiwan, and Hong Kong were 13.8, 15.9, and 19.9 per 100,000 people, respectively. The number of APE patients per year at our hospital tended to increase over the last decade which is similar to the previous studies in any area of the world including Asian Countries [2,13,14,15]. These may be due to the increase in physician awareness of the disease and fast accessibility to advanced diagnostic imaging. From chart reviews, we found that our physicians seldom use clinical predictability scores such as the Wells score to assess the probability of pulmonary embolism before sending the patients to CTPA. However, from a retrospective assessment of clinical predictability score, There were about 42.5% of APE cases have a Wells score ≤ 4 (PE unlikely) from a two-level Wells score assessment. Wells score used alone had a low predictive ability to exclude acute pulmonary embolism events. Unfortunately, D-dimer testing in our study data was not used for pretest assessment, most D-dimer testing was done after the diagnosis of APE was confirmed.

The clinical presentations were non-specific which dyspnea either at rest or exertion, oxygen desaturation, and tachycardia are the most common symptoms and signs. Extremities swelling suggestive of DVT was found in 18.3% with more common in the legs, while pain on limb palpation was found in only 5.8%. Concurrent ultrasonography for DVT was done in 292 patients (42.0%), 54.8 % of whom were found with concurrent DVT. Most patients (80.5%) had at least one risk factor. Active cancers, in which adenocarcinoma was the most common cell type, were the most common risk factor which is comparable to other previous Thai studies (21.1–62.1%) [16,17]. Metastatic stage of cancer was the strongest predictor of VTE due to increased thrombogenesis from the aggressiveness of tumor cells [18]. The incidence of cholangiocarcinoma, the second most common cancer associated with PE in our findings is comparable to the study from Khon Kaen, Thailand (19.5%) [17], but different from the Western study [19] because Thailand is the country with the highest incidence of cholangiocarcinoma in the world [20]. About one-third of our patients with PE were incidental PE during the workup for other diseases and cancer staging. Patients with incidental PE had comparable outcomes to symptomatic PE [21]. Hence, high awareness of APE especially in cancer patients, may lead to the proper investigation and early management. The utility and safety of prophylactic anticoagulation might be considered in this patient’s group. In our study, 141 patients (20.3%) had a history of major surgery or trauma requiring endotracheal or epidural anesthesia within the last 4 weeks before APE diagnosis. All of these patients group did not receive thromboembolic prophylaxis, proper thromboembolic prophylaxis strategy should be appropriate in selected patients.

The therapeutic measures of our patients are in the same practice as in Western countries. LMWH was used more often than UFH in acute treatment, except in patients who have creatinine clearance less than 30 mL/min and in patients who have more acute severe conditions. For long-term treatment, LMWH and warfarin were mostly used in the same proportion. Almost all of the LMWH used in long-term treatment in our study was used in cancer-associated PE. DOACs were used in cancer-associated PE and in patients who had co-administrative drug interaction with warfarin. Medical thrombolytic therapy with thrombolytic agents and surgical pulmonary thromboembolectomy was done in selected cases of high-risk PE, and most of them had favorable outcomes. Major bleeding from treatments was found at 8.2% but no fatal bleeding events.

Although the number of APE cases in a ten-year study period was rising, PE-related death tended to decline. These findings were similar to the study of the Chinese population during 1997–2008 [22]. In our study, the 30-day all-cause mortality rate was 19.1%, which is comparable to in-hospital death from previous Thai studies with a range from 15.5 to 21.3% [16,17], but higher than studies in Europe and North America that found all-cause mortality rate with a range from 8.7–17.4% [4,23]. Causes of death were mostly pneumonia, sepsis, and advanced cancers. PE-related mortality was only 5.6%. We observed that the high-risk PE mortality rate in our study was 29.5% which lower than the results from a study in the U.S., Stein et al. [24] found that Nineteen-Year trends in mortality of hospitalized patients in the United States with high-risk PE were decreased from 72.7% in 1999 to 49.8% in 2017. The lower death rate of high-risk PE in our study might be from rapid detection and rapid treatment with medical thrombolysis or surgical pulmonary thromboembolectomy.

To the best of our knowledge, this is the largest study in Thailand that reviewed clinical characteristics, risk factors, and outcomes of APE patients which may represent the Asian population. The limitation is the retrospective study in one center and some clinical data was not completed in all patients. A prospective study will be helpful to set proper management guidelines for this serious condition in the Thai and Asian populations.

## 5. Conclusions

APE was not uncommon in the Asian population. The trend of prevalence and mortality, and clinical characteristics were not different from Western countries. Active cancer, especially lung cancer and cholangiocarcinoma, and immobilization were the most common risk factors. The number of cases was on the rising trend, but PE-related death tended to decline. High-risk and intermediate-high-risk PE were associated with high mortality. Risk stratification and prompt management are warranted to improve outcomes.

## Figures and Tables

**Table 1:** Patient Characteristics and Co-morbidities.

Characteristics (*n* = 696)	Mean ± SD or *n* (%)
Age (years)	57.7 ± 15.7 (range 15–98)
**Sex**	
Male sex	286 (41.1)
Female sex	410 (58.9)
Suspected APE	468 (67.2)
Incidental APE	228 (32.8)
**Co-morbidities**	388 (55.7)
Hypertension	305 (43.8)
Diabetes mellitus	114 (16.4)
Renal diseases	69 (9.9)
Chronic obstructive pulmonary disease	38 (5.5)
Cirrhosis	31 (4.5)
CAD with prior myocardial infarction	30 (4.3)
Other chronic lung problems	28 (4.0)
Hematologic diseases	25 (3.6)
Connective tissue disease	22 (3.2)
Thalassemia	20 (2.9)
Obstructive sleep apnea	15 (2.2)
Obesity (BMI ≥ 30 kg/m^2^)	12 (1.7)
Nephrotic syndrome	11 (1.6)
Splenectomy	10 (1.4)
HIV infection	9 (1.3)
Vasculitis	3 (0.4)
Pregnancy	2 (0.3)
Ulcerative colitis	1 (0.1)
Active smoking	24 (3.4)
Chronic alcohol drinking	24 (3.4)
**Medications**	
Current antiplatelet	62 (8.9)
Current anticoagulant	35 (5.0)
**Venous thromboembolism**	
Current DVT proven by imaging	245 (35.3)
Prior PE > 3 months	12 (1.7)
Prior PE < 3 months	4 (0.6)
Previous DVT, PE	45 (6.5)
**Wells score, 2-level**	4.49 ± 2.54
Wells score ≤ 4	296 (42.5)
Wells score > 4	400 (57.5)
**Wells score, 3-level**	
Wells score < 2	144 (20.7)
Wells score 2–6	401 (57.6)
Wells score > 6	151 (21.7)

Note: Data are mean ± SD or *n* (%). Abbreviations: BMI, body mass index; CAD, coronary artery disease; HIV, Human immunodeficiency virus; APE, pulmonary embolism; DVT, deep vein thrombosis.

**Table 2:** Symptoms and Physical Findings.

Characteristics (*n* = 696)	Mean ± SD or *n* (%)
**Symptoms**	
Dyspnea	
at rest	453 (65.3)
at exertion	274 (39.5)
Cough	162 (23.3)
Respiratory failure (on invasive MV)	129 (18.6)
Chest pain	115 (16.5)
substernal	64/115 (55.7)
pleuritic	44/115 (38.2)
angina-like	7/115 (6.1)
Syncope	54 (7.8)
Palpitation	52 (7.5)
Hemoptysis	38 (5.5)
Dizziness	33 (4.8)
Fever	27 (3.9)
Altered mental status	23 (3.3)
Cyanosis	11 (1.6)
Diaphoresis	9 (1.3)
**Physical findings**	
Oxygen desaturation (SpO_2_ < 90%)	333 (48.3)
Room air SpO_2_ (%)	89.4 ± 7.9 (38–100)
Tachycardia (HR > 110/min)	326 (46.9)
HR (beats/min)	105.4 ± 18.4 (46–160)
Tachypnea (RR ≥ 30/min)	158 (22.7)
RR (breaths/min)	24.3 ± 6.1 (14–60)
Extremity swelling suggestive of DVT	127 (18.3)
leg	104/127 (88.9)
thigh	9/127 (7.7)
upper extremity	4/127 (3.4)
Pain on limb palpation	40 (5.8)
Low BP (<90/60 mmHg)	83 (11.9)
SBP (mmHg)	116.4 ± 21.9 (50–200)
DBP (mmHg)	72.3 ± 13.9 (20–130)
Rales (crackles)	142 (20.4)
Decreased breath sounds	110 (15.8)
Wheeze & rhonchi	37 (5.3)
Increased P2	26 (3.7)
Right ventricular lift	23 (3.3)
Jugular venous distension	22 (3.2)

Note: Data are mean ± SD or *n* (%). Abbreviations: BP, blood pressure; DBP, diastolic blood pressure; DVT, deep vein thrombosis; HR, heart rate; MV, mechanical ventilation; P_2_, second pulmonic heart sound; RR, respiratory rate; SBP, systolic blood pressure; SpO_2_, peripheral capillary oxygen saturation.

**Table 3:** Management of Patients with APE.

Characteristics (*n* = 696)	*n* (%)
**Acute treatment**	
Heparin	
LMWH alone	403 (57.9)
UFH alone	127 (18.2)
UFH + LMWH	104 (14.9)
Fondaparinux	4 (0.6)
Thrombolytic therapy	37 (5.3)
Surgical thromboembolectomy	12 (1.7)
IVC filter	14 (2.0)
Supportive care	11 (1.6)
No treatment due to contraindication	25 (3.6)
Denied treatment	9 (1.3)
**Long term treatment**	
Warfarin	252 (36.2)
LMWH	247 (35.5)
DOACs	40 (5.7)

Note: Data are *n* (%). Abbreviations: IVC, inferior vena cava; LMWH, low molecular weight heparin; NA, not applicable; DOACs, direct oral anticoagulants; UFH, unfractionated heparin.

**Table 4:** Outcomes of Patients with Acute Pulmonary Embolism (*n* = 696).

Outcomes	Median (IQR) or *n* (%)
30-day all-cause mortality	133 (19.1)
30-day PE-related mortality	39 (5.6)
30-day non-PE-related mortality	94 (13.5)
Sepsis	37 (39.4)
Pneumonia	25 (26.6)
Cancer	11 (11.7)
Septic shock	10 (10.6)
Others	11 (10.7)
Major bleeding	57 (8.2%)
UGIH	34 (4.9)
ICH	11 (1.6)
LGIH	8 (1.1)
Hemoptysis	4 (0.6)
Recurrent PE within 14 days	2 (0.3)
Hemodynamic collapse	37 (5.3)
Mechanical ventilation (MV) support	158 (22.7)
MV days (median, IQR)	4 (2, 10)
Vasopressor	90 (12.9)
Turn to CTEPH	25 (3.6%)
Hospital stay (days) (median, IQR)	12 (6, 23)

Note: Data are median, IQR, or *n* (%). Abbreviations: CTEPH, chronic thromboembolic pulmonary hypertension; ICH, intracerebral hemorrhage; LGIH, lower gastrointestinal hemorrhage; PE, pulmonary embolism; UGIH, upper gastrointestinal hemorrhage.

## Data Availability

The data that support the findings of this study are available on request from the corresponding author.

## Supplementary Materials

Published online alongside the manuscript can be downloaded at: https://www.mdpi.com/article/10.3390/jcm11236954/s1, Table S1: Risk Factors of Acute Pulmonary Embolism; Table S2: Cancer-associated Acute Pulmonary Embolism; Table S3: Electrocardiogram Findings at Presentation; Table S4: Chest X-ray Findings at Presentation; Table S5: Computed Tomography (CT) Findings of APE; Table S6: Other Investigations Including Serum Biomarkers, Ultrasound DVT, and Echocardiography.
