# Supplementary material for: Clinical Characteristics, Risk Factors, and Outcomes of Acute Pulmonary Embolism in Asian Population
Source: J Clin Med. 2022 Nov 25;11(23):6954. doi: 10.3390/jcm11236954 (PMC9741370; doi:10.3390/jcm11236954)
Supplement: Supplementary file 1 [file jcm-11-06954-s001.zip › jcm-2044392-supplementary.pdf]

Table S1 Risk Factors of Acute Pulmonary Embolism

**Characteristics (*n* = 696)**	***n* (%)**
**No risk factor**	136 (19.5)
**Risk factors**	560 (80.5)
Active malignancy	388 (55.7)
Immobility – total body immobilization	211 (30.4)
Surgery or trauma requiring endotracheal intubation or epidural anesthesia within the last 4 weeks	141 (20.3)
Oral contraceptives/ estrogen therapy	27 (3.9)
Indwelling venous catheter	23 (3.3)
Recent significant trauma	12 (1.7)
Long travel history > 6 h	10 (1.4)
Right side endocarditis	2 (0.3)
Protein C deficiency	31 (4.4)
Protein S deficiency	12 (1.7)
Antithrombin III deficiency	6 (0.9)
Lupus anticoagulant	15 (2.1)
Antiphospholipid	2 (0.3)

Note: Data are n (%).

Table S2 Cancer-associated Acute Pulmonary Embolism

**Characteristics**	***n* (%)**
**Primary malignancy** (*n*=388)	
Lung cancer	98 (25.3)
Cholangiocarcinoma	67 (17.3)
Colorectal cancer	38 (9.8)
Hepatocellular carcinoma	26 (6.7)
Lymphoma	25 (6.4)
Ovarian cancer	24 (6.2)
Stomach cancer	16 (4.1)
Sarcoma	14 (3.6)
Pancreatic cancer	12 (3.1)
Breast cancer	11 (2.8)
Cervical cancer	9 (2.3)
Endometrium carcinoma	9 (2.3)
Brain tumor	7 (1.8)
Multiple myeloma	5 (1.3)
Acute leukemia	4 (1.0)
Neuroendocrine tumor	4 (1.0)
Unknown primary site	4 (1.0)
Urinary bladder cancer	3 (0.8)
Prostate cancer	3 (0.8)
Thyroid cancer	3 (0.8)
Gall bladder cancer	3 (0.8)
Others	3 (0.8)
**Cell type (*n*=209)**	
Adenocarcinoma	147 (70.3)
Squamous cell carcinoma	18 (8.6)
Sarcoma	14 (6.7)
Diffuse large B cell lymphoma	13 (6.2)
Others	17 (8.1)
**Distant metastases**	282 (72.7)

Note: Data are n (%).

Table S3 Electrocardiogram Findings at Presentation

**Characteristics (*n* = 696)**	***n* (%)**
Sinus tachycardia	393 (56.5)
S1Q3T3	160 (23.0)
Right ventricular strain pattern	112 (16.1)
Shift of transitional zone to V5	47 (6.8)
Peripheral low voltage	47 (6.8)
Non-specific ST segment and T wave changes	42 (6.0)
Right axis deviation	39 (5.6)
ST depression I, II, V4-V6	26 (3.7)
Q in III, aVF but not in lead II	23 (3.3)
Atrial fibrillation	23 (3.3)
Dominant R wave in V1	20 (2.9)
Complete RBBB	19 (2.7)
Incomplete RBBB	19 (2.7)
Left axis deviation	14 (2.0)
ST elevation at aVR >1 mm	9 (1.3)
ST elevation in at least one of the leads III, aVR, and V1–V4	7 (1.0)
Atrial flutter	5 (0.7)
Atrial premature contractions	4 (0.6)
ST elevation I, II, V4-V6	4 (0.6)
P pulmonale 2.5 mV in lead II	3 (0.4)
ST elevation in leads III and/or V1/V2 with concomitant ST depression in leads V4/V5–V6	3 (0.4)
Atrioventricular block	1 (0.1)

Note: Data are n (%). Abbreviations: RBBB, right bundle branch block

Table S4 Chest X-ray Findings at Presentation

**Characteristics (*n*=696)**	***n* (%)**
Normal	327 (47.1)
Abnormal from underlying conditions	193 (29.8)
Pleural effusion	137 (19.7)
Infiltration	126 (18.1)
Westermark sign	100 (14.4)
Cardiomegaly	82 (11.8)
Prominent central pulmonary trunk	53 (7.6)
Hampton hump	46 (6.6)
Atelectasis	20 (2.9)
Elevated hemidiaphragm	9 (1.3)

Note: Data are n(%).

Table S5 Computed Tomography (CT) Findings of APE

**Characteristics (*n* = 696)**	***n* (%)**
**Site of pulmonary embolism**	
Mediastinal (central)	310 (44.5)
Lobar	195 (28.0)
Segmental	577 (82.9)
Subsegmental	112 (16.1)
Pulmonary infarction	152 (21.8)
**Signs of RV pressure load**	
IVS flattened	211 (30.3)
RV/LV > 1	190 (27.3)
Contrast media reflux into IVC	189 (27.2)
IVS bowing	79 (11.4)

Note: Data are n (%). Abbreviations: IVC, inferior vena cava; IVS, interventricular septum; LV, left ventricular; RV, right ventricular.

Table S6 Other Investigations Including Serum Biomarkers, Ultrasound DVT, and Echocardiography

**Characteristics**	**Median (IQR) or *n* (%)**
**Serum biomarkers**	
Troponin-T (*n*=228)	
Median (IQR)	53.1 (19.6, 159.5)
D-dimer (*n*=129)	
Median (IQR)	8,850.0 (4,242.5, 18748.5)
Lactate (*n*=88)	
Median (IQR)	2.8 (1.7, 4.8)
**Ultrasound DVT (*n*=292)**	
Positive	160 (54.8)
popliteal vein	91 (56.9)
femoral	42 (26.3)
superficial femoral vein	18 (11.3)
arm vein	3 (1.9)
other	6 (3.8)
Negative	132 (45.2)
**Echocardiogram findings (*n*=332)**	
No RV dysfunction	145 (43.7)
RV dysfunction	132 (39.8)
RV/LV > 1	101 (30.4)
McConnell's sign	65 (19.6)
TAPSE <1.6 cm	18 (5.4)
RV hypokinesia	18 (5.4)
RV thrombus	11 (3.3)
Leftward shifting IVS	3 (0.9)

Note: Data are median, IQR, or n (%). Abbreviations: DVT, deep vein thrombosis; IVS, interventricular septum; LV, left ventricular; RV, right ventricular; TAPSE, *tricuspid annular plane systolic excursion*.
